# Surface-tailoring of emulsomes for boosting brain delivery of vinpocetine via intranasal route: *in vitro* optimization and *in vivo* pharmacokinetic assessment

**DOI:** 10.1080/10717544.2022.2110996

**Published:** 2022-08-16

**Authors:** Hibah M. Aldawsari, Shaimaa M. Badr-Eldin, Nourah Y. Assiri, Nabil A. Alhakamy, Anna Privitera, Filippo Caraci, Giuseppe Caruso

**Affiliations:** a Department of Pharmaceutics, Faculty of Pharmacy, King Abdulaziz University, Jeddah, Saudi Arabia; b Center of Excellence for Drug Research and Pharmaceutical Industries, King Abdulaziz University, Jeddah, Saudi Arabia; c Mohamed Saeed Tamer Chair for Pharmaceutical Industries, King Abdulaziz University, Jeddah, Saudi Arabia; d Advanced Drug Delivery Research Group, Faculty of Pharmacy, King Abdulaziz University, Jeddah, Saudi Arabia; e Department of Drug and Health Sciences, University of Catania, Catania, Italy; f Unit of Neuropharmacology and Translational Neurosciences, Oasi Research Institute - IRCCS, Troina, Italy

**Keywords:** Vinpocetine, surface-tailored intranasal emulsomes, cationization, PEGylation, pharmacokinetics, brain delivery

## Abstract

Vinpocetine (VNP), a semisynthetic active pharmaceutical ingredient, is used for oral management of cerebrovascular diseases because of its ability to enhance the blood flow to the brain. However, despite that, the therapeutic application of VNP is restricted due to its reduced bioavailability and diminished brain levels that could be attributed to its low aqueous solubility, short half-life, and presystemic metabolism exposure. Accordingly, the goal of this work was to explore the ability of surface-tailored intranasal emulsomes to boost brain delivery of the drug. A 3^2^2^1^ factorial design was implemented to explore the impact of phospholipid (PL) to solid lipid weight ratio, PL to cholesterol molar ratio, and type of solid lipid on vesicle size, zeta potential, drug entrapment, and release efficiency of the new developed VNP emulsomes. Tailoring of the optimized emulsomal surface formulation was performed using either cationization or PEGylation approaches to boost blood–brain barrier penetration. The pharmacokinetic assessment in rats showed significantly improved bioavailability of VNP emulsomal formulations compared to the oral market product. Additionally, surface-tailored emulsomes exhibited significantly higher brain levels compared to the optimized emulsomes. Based on these findings, the proposed surface-tailored emulsomes could be considered as a promising platform for achieving high brain levels of VNP following intranasal administration.

## 1. Introduction

Central nervous system (CNS) disorders represent a major public health challenge (Thakur et al., 2016; Caruso et al., 2022). As per World Health Organization (WHO) statistics (www.who.int; accessed on May 24), over than 1 billion people are diagnosed for neurological disorders worldwide. The blood–brain barrier (BBB) permeability and specificity represent crucial challenges for drug delivery to the brain in safe and adequate manner (Liu & Jiang, 2022). The physiological and histological structure of the BBB could be regarded as the main factor accounting for its limited permeability (Fresta et al., 2020). The tight junctions part of the cerebral capillary endothelium along with the transporters play a key role in restricting the delivery of drugs to the CNS (Zidan & Aldawsari, 2015; Vieira & Gamarra, 2016).

The intranasal route represents one of the most effective ways to convey drugs to the brain, especially thanks to its ability to circumvent BBB via olfactory and trigeminal routes (El-Zaafarany et al., 2016; Erdő et al., 2018). Being a noninvasive route that offers rapid onset of action and surpasses presystemic metabolism, nasal route has become one of the most attractive administrations that target brain delivery (Harbi et al., 2016).

Nanosized lipid-based vesicles have been explored as promising platform for drugs’ delivery to brain upon nasal administration (Hong et al., 2019; Grasso et al., 2021). To promote brain targeting of such systems, several approaches have been proposed including surface cationization, surface tailoring by targeting ligands, as well as triggering the drug release by magnetic field, temperature, ultrasound, or any other external factor (Harbi et al., 2016; Vieira and Gamarra, 2016; Nageeb El-Helaly et al., 2017).

Emulsomes are lipoidal vesicles comprising solid lipid (SL) core enclosed by a phospholipid (PL) multilayer sheath. It combines advantageous characteristics of both nanoemulsions and liposomes (Pal et al., 2012; Awan et al., 2020). The PL layers, being the outermost structure of emulsomes, eliminate the need of surfactant for stabilization of the emulsomes, thus, infer a high level of biocompatibility for clinical applications. In comparison to liposomal vesicular systems, the PL sheath enhances the solubility and biological availability of sparingly soluble drugs. A featured property of emulsomes is the existence of the lipid core as solid or liquid crystalline state rather than being oil in a liquid state (Fahmy et al., 2020). This feature distinguishes emulsomes from nanoemulsions and allows them to entrap higher amounts of lipid soluble drugs with extended release profile. The site specificity of emulsomes gives to this unique delivery system a merit over liposomes and emulsions; the nanosize could effectively boosts drug targeting effect of the emulsomal dispersions (Gupta & Vyas, 2007; El-Zaafarany et al., 2016). It is worthy to note that the similarity of the shell structure of both liposomes and emulsomes directed the attention of researchers to investigate the tailoring of emulsomal surface to meet specific criteria such as enabling drug targeting. Amongst the approaches for emulsomal surface modification is the use of macrophage specific ligands, monoclonal antibodies, or crystalline bacterial cell surface layer (S-layer) proteins (Gupta & Vyas, 2007; Ucisik et al., 2015). Cationization has also been explored for emulsomal surface tailoring with the aim to target an antiviral drug to liver cells (Vyas et al., 2010). However, no studies have investigated the emulsomal surface tailoring for brain targeting.

Vinpocetine (VNP; 14-ethoxycarbonyl-(3a,16a-ethyl)-14,15-eburnamine) is a synthetic derivative of vincamine alkaloid used for the management of several CNS disorders including cerebrovascular ischemia, Alzheimer’s disease, and other different types of dementia (Zhang et al., 2018). Unfortunately, VNP is a poorly aqueous soluble active agent that is exposed to dramatic presystemic metabolism, therefore, it possesses an extremely short half-life. These faults could result in reduced bioavailability and diminished brain concentrations restricting its clinical applications (Vyas et al., 2010; Ucisik et al., 2015). It is then highly recommended to develop a drug delivery system able to effectively enhance VNP solubility and promote its brain delivery. Based on the above, this work aimed at developing and optimizing intranasal surface-tailored VNP nanoemulsomes for boosting drug brain levels.

## 2. Materials and methods

### 2.1. Materials

VNP, cholesterol (CH), tristeararin (TS), tripalmitin (TP), stearylamine (SA), and cellophane membrane (MWCO 12000 − 14000 Da) were obtained from Sigma-Aldrich (St. Louis, Missouri, USA). Lipoid S 100® (L-α-Phosphatidylcholine from soya) and [N-(carbonyl-methoxypropylethyleneglycol-2000)-1,2-distearoyl-sn-glycero-3-phosphoethanolamine, sodium salt] (MPEG-DSPE) were a gift from Lipoid GMBH (Ludwigshafen, Germany). All the remaining chemicals/materials were of analytical grade and obtained from Thermo Fisher Scientific Inc. (Pittsburgh, PA, USA) unless specified otherwise.

### 2.2. Preparation of VNP emulsomes

The previously reported modified thin film hydration method of Paliwal et al. (2009) was employed for preparing emulsomes. Sixty milligrams of VNP were dissolved with specified amounts of PL, SL in 10 mL of chloroform/methanol blend (2:1, v/v). Rotavapor R-200 (BÜCHI Labortechnik AG, Swizerland) was used to evaporate the solvent blend at 40 °C. The formed residual films were placed in a vacuum oven (Model 6505, Thermo Fisher Scientific, USA) to remove the residual traces of organic solvent. The films were agitated gently with specified volume of phosphate buffer (PBS) at pH 6.8 for 60 min at room temperature. The formed dispersion was subjected to ultrasonication for two cycles (90 s each) with 4 min between cycles (Ding et al., 2015; El-Zaafarany et al., 2016). The developed emulsomes were maintained at 4 °C until peforming further studies.

### 2.3. Vesicle size and zeta potential

Zetasizer Nano S (Malvern instrument Ltd, UK) was employed to measure the mean vesicle size (VS) and zeta potential (ZP) of VNP emulsomes (*n* = 6). Emulsomes were diluted appropriately prior to measurement.

### 2.4. Entrapment efficiency

Entrapment efficiency (EE%) of VNP loaded emulsomes was determined indirectly. Emulsomal dispersions were subjected to ultracentrifugaiton at 100,000 rpm for 60 min at 4 °C for separation of unentrapped VNP (OptimaTM MAX-XP, Beckman Coulter Inc., USA). PBS at pH 6.8 was used for residue washing; the residue was subjected to ultracentrifugation again for 60 min. PBS at pH 6.8 was used to appropriately dilute the mingled supernatant prior to quantification of drug using a UV spectrophotometer (UV-2600 PC, Shimadzu Corporation, Kyoto, Japan) at *λ*_max_ 273 nm (Paliwal et al., 2009; Zidan & Aldawsari, 2015; El-Zaafarany et al., 2018). Each determination was done in triplicate. The EE% was calculated applying the following equation:

$$\mathrm{EE}\%=\{\frac{\left(\;Dt-Df\right)}{Dt}\;\}\times \;100$$

where *Dt* and *Df* represent the amount of total drug and unentrapped drug, respectively.

#### 2.4.1. In vitro release

Glass basket dialysis technique was employed for studying *in vitro* release using modified USP dissolution tester (Type II, DT 720 Series, Erweka GmbH, Germany) (Panwar et al., 2010; Narayan et al., 2016). The release medium used was 200 mL PBS at pH 6.8. Emulsomal formulation samples (equivalent to 0.5 mg of drug) were placed in glass cylindical tubes. A dialysis cellulose membrane was used to cover on end of the tube, while the other end was hanged to the dissolution tester shaft that was then emerged in the dissolution apparatus vessel containing the release medium. The apparatus operated at 35 ± 0.5 °C with a rotation speed of 50 rpm to simulate nose mixing conditions (El-Zaafarany et al., 2016). The vessels were covered throghout the experiment to reduce medium loss by evaporation. Withdrawal of aliquots was performed at time points ranging from 0.25 to 24 h. VNP released was quantified using a previously reported HPLC method with UV detection at 273 nm (Ding et al., 2015). With regard to the release of the pure drug (VNP), it is reported to have low solubility at pH 6.8 (about 2.44 µg/mL) and consequently a low release percentage (Ding et al., 2015).

### 2.5. 3^2^2^1^ Full factorial design for VNP emulsomes optimization

3^2^2^1^ full factorial design was used to explore the influence of formulation variables on the emulsomal characteristics. The independent variables studies were PL:SL weight ratio; X_1_, PL:CH molar ratio; X_2_, and SL type; X_3_. X_1_ and X_2_ were studied at three levels: (1:1, 2:1, and 3:1 w/w) for X_1_, (2:1, 4:1, and 8:1 mole/mole) for X_2,_ while X_3_ was explored at two levels (TS and TP). Eighteen runs were generated as per the design (E1-E18), while six runs were prepared for the optimized with surface modification (E19-E24) (Table 1).

**Table 1.** Combination of independent variables in VNP emulsomes experimental runs prepared according to 3^2^2^1^ full factorial design and their corresponding responses.

Run	PL:SL (X1)	PL:CH (X2)	SL type (X3)	VS (nm) ± SD*	ZP (mV) ± SD*	EE (%) ± SD**	RE24h (%) ± SD**
**E1**	1:1	2:1	TP	149.95 ± 5.59	–33.88 ± 5.20	71.70 ± 3.82	25.60 ± 2.30
**E2**	2:1	2:1	TP	254.80 ± 0.28	–42.54 ± 3.45	76.00 ± 5.66	29.71 ± 1.83
**E3**	3:1	2:1	TP	258.30 ± 3.11	–51.74 ± 3.73	78.60 ± 1.98	38.80 ± 2.32
**E4**	1:1	4:1	TP	376.50 ± 3.54	–42.60 ± 4.53	74.80 ± 1.13	35.11 ± 2.81
**E5**	2:1	4:1	TP	284.75 ± 3.89	–61.12 ± 2.01	77.00 ± 1.41	42.90 ± 3.91
**E6**	3:1	4:1	TP	450.00 ± 1.40	–66.00 ± 5.66	80.70 ± 2.40	46.63 ± 4.84
**E7**	1:1	8:1	TP	333.00 ± 4.24	–54.00 ± 5.93	76.00 ± 4.24	42.62 ± 4.40
**E8**	2:1	8:1	TP	349.00 ± 1.41	–63.92 ± 2.16	78.50 ± 3.54	50.01 ± 3.12
**E9**	3:1	8:1	TP	368.00 ± 4.24	–71.92 ± 3.99	82.00 ± 4.24	54.73 ± 8.71
**E10**	1:1	2:1	TS	156.70 ± 2.55	–42.21 ± 3.27	72.40 ± 2.26	21.00 ± 4.01
**E11**	2:1	2:1	TS	318.01 ± 4.95	–44.00 ± 4.95	74.00 ± 5.94	24.02 ± 4.41
**E12**	3:1	2:1	TS	329.50 ± 3.54	–48.50 ± 4.95	78.00 ± 2.83	25.62 ± 2.15
**E13**	1:1	4:1	TS	532.52 ± 3.54	–46.00 ± 4.53	75.00 ± 3.25	28.23 ± 2.91
**E14**	2:1	4:1	TS	554.54 ± 2.12	–64.00 ± 1.90	76.00 ± 3.25	29.83 ± 3.40
**E15**	3:1	4:1	TS	676.11 ± 4.24	–61.00 ± 2.26	79.50 ± 1.13	34.20 ± 4.61
**E16**	1:1	8:1	TS	824.53 ± 0.71	–58.01 ± 3.78	76.80 ± 1.84	46.24 ± 5.61
**E17**	2:1	8:1	TS	857.20 ± 4.24	–72.03 ± 2.83	79.40 ± 5.80	47.00 ± 5.63
**E18**	3:1	8:1	TS	952.31 ± 2.83	–78.01 ± 2.83	83.00 ± 3.11	49.70 ± 6.61
**E19**	3:1	2:1	TS	433.83 ± 24.28	11.83 ± 1.76	79.50 ± 6.76	22.84 ± 3.74
**E20**	3:1	2:1	TS	484.00 ± 43.69	13.00 ± 1.00	83.33 ± 11.31	29.33 ± 3.80
**E21**	3:1	2:1	TS	521.00 ± 24.26	14.40 ± 2.05	75.59 ± 4.91	30.64 ± 1.60
**E22**	3:1	2:1	TS	194.67 ± 27.21	–30.30 ± 2.46	81.39 ± 1.86	19.45 ± 1.64
**E23**	3:1	2:1	TS	202.00 ± 22.69	–22.20 ± 2.71	88.45 ± 6.09	18.18 ± 1.75
**E24**	3:1	2:1	TS	229.00 ± 15.77	–17.80 ± 2.99	93.45 ± 3.11	14.75 ± 3.69

Abbreviations: *SD*: Standard deviation; VNP: vinpocetine; PL:SL: phospholipid:solid lipid weight ratio; PL:CH: phospholipid:cholesterol molar ratio; SL: solid lipid; TP: tripalmitin; TS: tristearin; VS: vesicle size; ZP: zeta potential; EE%: entrapment efficiency %; RE_24h_: release efficiency after 24 h. **E12 = **optimized VNP emulsomes; **E19-E21 = **stearylamine surface-tailored emulsomes, **E19 **=** **0.25 M; **E20 **=** **0.5 M; **E21 **=** **0.75 M; **E22-E24 = **MPEG-DSPE surface-tailored emulsomes, **E22 **=** **0.1 M; **E23 **=** **0.2 M; **E24 **=** **0.3 M. Results are presented as mean ± *SD*, *n = 5, **n = 3.

VS (nm; Y_1_), ZP (mV; Y_2_), EE (%; Y_3_), and RE at 24 h (%; Y_4_) were investigated as responses and Design-Expert software (version 12; Stat-Ease, Inc., Minneapolis, MN, USA) was used for statistical data analysis. The model with the best-fit value of each parameter was chosen for each dependent variable. Analysis of variance (ANOVA) was employed to assess the significance of the independent variables on the responses and the interaction between them at 95% significance level. Further, the optimized VNP emulsomes were selected based on the desirability function. The criteria set for optimized emulsomes were minimized VS and RE_24h_, and maximized absolute ZP value and EE%.

### 2.6. Surface tailoring of optimized VNP emulsomal formulation

Surface tailoring of optimized VNP emulsomal formulation was performed using either SA, as cationic charge inducer, or MPEG-DSPE as PEGylated PL at various molar ratio. The modifier was dissolved in 10 mL chloroform/methanol mixture (2:1, v/v) along with drug and emulsomal constituents. The preparation was completed by using the previously mentioned method described in subsection 2.2 (Paliwal et al., 2009; El-Laithy et al., 2011). The surface-tailored optimized emulsomes were further characterized for non-tailored emulsomes as previously mentioned.

### 2.7. Morphological studies

The shape of the optimized emulsomal formulation as well as its corresponding selected SA and MPEG-DSPE surface-tailored formulations were subjected to inspection by using transmission electron microscope (TEM, TitanTM D3187, FEI Company, Thermo Fisher Scientific, USA). The emulsomal formulation was applied after dilution to a carbon coated grid and allowed for adsorption for 2 min. Staining of the emulsomal dispersion was then performed by using phosphotungsitc acid. The grid was allowed for air-drying after excess stain removal. The samples were then visualized with magnification power of 22500X (El-Zaafarany et al., 2016).

### 2.8. *In vivo* evaluation

*In vivo* pharmacokinetic evaluation of the chosen optimized VNP emulsomes and their corresponding surface-tailored emulsomes was performed in rats. Male Wistar rats (*n* = 96; ∼250 g) were provided by the animal facility, King Abdulaziz University (KAU, Jeddah, Saudi Arabia). The study was approved by the Research Ethics Committee, Faculty of Pharmacy (Ref. # PH-109-41). The random distribution of rats among 4 groups was performed; Groups I, II, and III were composed by animals in which the new formulations were tested, while the animals of Group IV received the standard control. The rats involved in the study received VNP in a dose of 10 mg/kg (Ding et al., 2015): Group I = optimized VNP emulsomes (E12), Group II = SA surface-modifed VNP emulsomes (E19), Group III = MPEG-DSPE surface-tailored VNP emulsomes (E24), and Group IV = Vinporal® (market product). A specified volume of emulsomal formulation was instilled in each nostril using polyethylene tubes, while the market product was administered orally via intragastric tubing.

Collection of blood samples in heparinized tubes was performed via the orbital vein at preset time points for a period of 12 h. With regard to plasma separation, samples were centrifuged at 3000 rpm for 15 min. Six rats from each group were scarified at predetermined time intervals. The brain was washed by using ice-cold PBS at pH 7.4 after removal from the skull. Three-fold volumes of PBS at pH 7.4 was added to the washed brain, which was then subjected to homogenization at 10,000 rpm for 60 s (T18 basic high speed homogenizer, ULTRA TURRAX®, Brazil). Both plasma and brain homogenates were subjected to storage at −80 °C until analysis (El-Zaafarany et al., 2016).

To quantify VNP in plasma and brain homogenates, the method reported by Xia et al. employing liquid chromatography-tandem mass spectrometry (LC-MS/MS) was used (Xia et al., 2010).

### 2.9. Statistical analysis

Data were expressed as mean ± standard deviation (*SD*). Statistical analysis was performed by using GraphPad Prism software, version 8.3.0 (GraphPad Software, San Diego, CA). ANOVA followed by a *post hoc* test were used for multiple comparisons. Specifically, Two-way ANOVA followed by Sidak’s multiple comparisons were applied for statistical analysis of plasma concentrations. *C*_max_ and AUC were statistically analyzed using One-way ANOVA followed by Tukey’s multiple comparisons, while *T*_max_ was statistically analyzed using nonparametric Kruskal–Wallis test. The statistical significance was set at *p*-value ≤ .05.

## 3. Results and discussion

### 3.1. Fit statistics

The sequential model representing each response was chosen as per adequate agreement between predicted and adjusted *R*^2^. The VS and RE data fitted the two-factor interaction (2FI) model, while the ZP and EE data fitted the linear model. The predicted *R*^2^ for VS, ZP, EE, and RE were 0.9569, 0.8463, 0.9178, and 0.8976, respectively, while the adjusted *R*^2^ for the studied variables were 0.9910, 0.9032, 0.9483, and 0.9785, respectively. The lowest predicted residual sum of squares (PRESS) confirms the validity of the selected models (Table 2).

**Table 2.** Model fit statistics for the responses of VNP emulsomes prepared according to 3^2^2^1^ full factorial design.

Response	Model	*p*-value	*R* ^2^	Adjusted *R* ^2^	Predicted *R* ^2^	Adequate precision	PRESS	Significant factors and interactions
**Y_1_**: VS (nm)	2FI	.0001	0.9979	0.9910	0.9569	39.170	41932.77	X_1_, X_2_, X_3_, X_1_X_2_, X_2_X_3_
**Y_2_**: ZP (mV)	Linear	<.0001	0.9317	0.9032	0.8463	18.831	405.18	X_1_, X_2_
**Y_3_**: EE (%)	Linear	<.0001	0.9635	0.9483	0.9178	25.039	13.31	X_1_, X_2_
**Y_4_**: RE_24h_ %	2FI	.0006	0.9949	0.9785	0.8976	24.557	191.22	X_1_, X_2_, X_3_, X_1_X_3_, X_2_X_3_

VNP: vinpocetine; VS: vesicle size; ZP: zeta potential; EE%: entrapment efficiency %; RE_24h_: release efficiency after 24 h; 2FI: two-factor interaction; PRESS: predicted residual sum of squares.

### 3.2. Effect on vesicle size (Y_1_)

**Figure 1.**
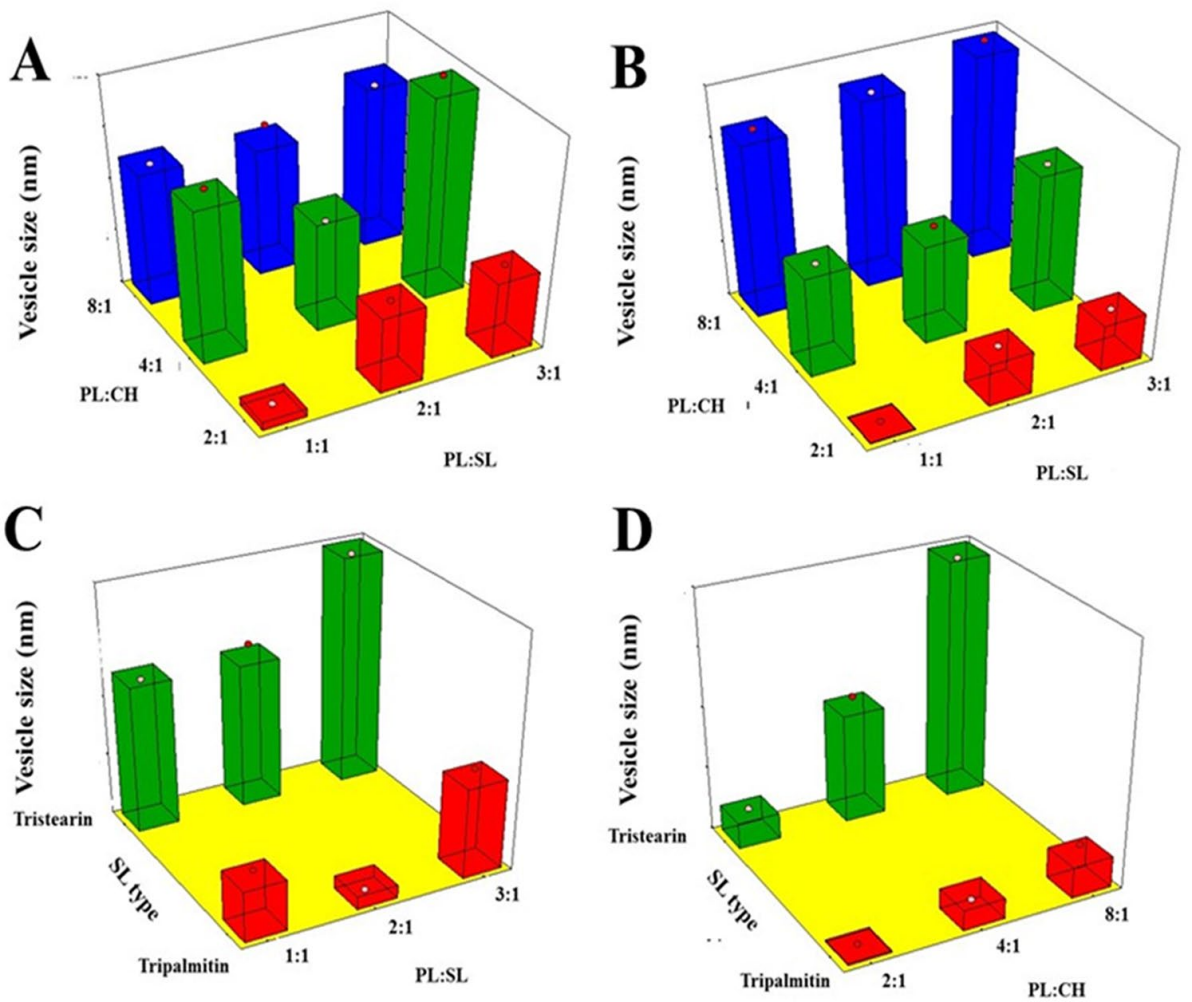
Response 3D surface plot for the influence of PL:SL weight ratio (X_1_), PL:CH molar ratio (X_2_), and SL type (X_3_) on VS (Y_1_) of VNP emulsomes. (A) SL type (Tripalmitin), (B) SL type (Tristearin), (C) PL:CH (4:1), and (D) PL:SL (2:1).

VS is one of the influential criteria that can exert an impact on drug entrapment, release, and cellular uptake or penetration via BBB (Banks et al., 2020). Moreover, it could affect the clearance mediated by the reticuloendothelial system (Tang et al., 2019). Reducing the VS leads to boosting the surface area and consequently drug absorption; in addition, nanosized systems could escape the uptake by the immune system (Xia et al., 2010; Danaei et al., 2018). Based on the results shown in Table 1, it was evident that although the size of all vesicles were within the nanorange, emulsomes prepared using TS exhibited larger size compared to those prepared using TP (Table 1). ANOVA, based on the 2FI polynomial model, revealed that the linear terms corresponding to the investigated variables had significant impact on VS (*p* = .0028 for X_1_ and *p* < .001 for X_2_ and X_3_). Additionally, the interaction terms X_1_X_2_ and X_2_X_3_, corresponding to the interaction between PL:CH and either PL:SL or SL type (X_2_X_3_) were also significant (*p* = .0323 and .0001, respectively). The three-dimensional surface plots presented in Figure 1 illustrate the impact of the studied independent variables on the VS response.

It was evident that VS increases upon increasing the PL:CH ratio. This observation could be attributed to the possible multiple bilayers formation that results in larger VS (El-Zaafarany et al., 2016). Vyas et al. have reported similar increase in size of emulsomes at higher molar ratio of PL relative to SL and CH prepared for liver delivery of zidovudine (Vyas et al., 2010). On the contrary, the relation between VS and CH showed inverse pattern, where reduction of CH ratio led to larger VS. This might be explained by the fact that high CH levels of impart higher lipophilic property to the developed emulsomes that hinder water uptake across lipid bilayer resulting in decreased size. This finding is in accordance with the results showed by Sudhakar et al. (2016), reporting an inverse relationship between VS and CH amount used in the formulation of stealth liposomes for delivery of ritonavir. Concerning SL type, emulsomes prepared using TS exhibited higher size compared to those prepared suing TP. TP and TS have very different melting points but very similar molecular structures (the fatty acid chains on tripalmitin (C16) differ from the fatty acid chains on tristearin (C18) by only two carbons) (Seetapan et al., 2010; Pizzirusso et al., 2018); the different size of these two triglycerides along with their different physicochemical properties could be responsible of the smaller vesicles TP-based compared to that with TS (Seetapan et al., 2010). Vyas et al. reported similar higher VS of TS-based vesicles relative to trilaurin-based ones (Vyas et al., 2010). Lastly, as shown by Scalia et al., a higher surfactant/lipid ratio could result in a reduction of the particle size, even though an opposite trend, which needs to be further investigated was observed for PL:SL (3:1) (vs. PL:SL (2:1)) (Scalia et al., 2015).

### 3.3. Effect on ZP (Y_2_)

ZP describes the surface charge of the vesicles and indicates their physical stability against aggregation. The vesicular dispersion is regarded as stable system for absolute ZP values greater than 30 mV owing to electrostatic repulsion that hinders vesicles clumping (Zhou & Chen, 2015). All the proposed systems were negatively charged as presented in Table 1. The absolute ZP values were greater than 30 mV indicating the stability of the vesicles against aggregation. According to ANOVA results, both PL:SL weight ratio (X_1_) and PL:CH molar ratio (X_2_) exhibited significant impact on ZP (*p* < .001).

**Figure 2.**
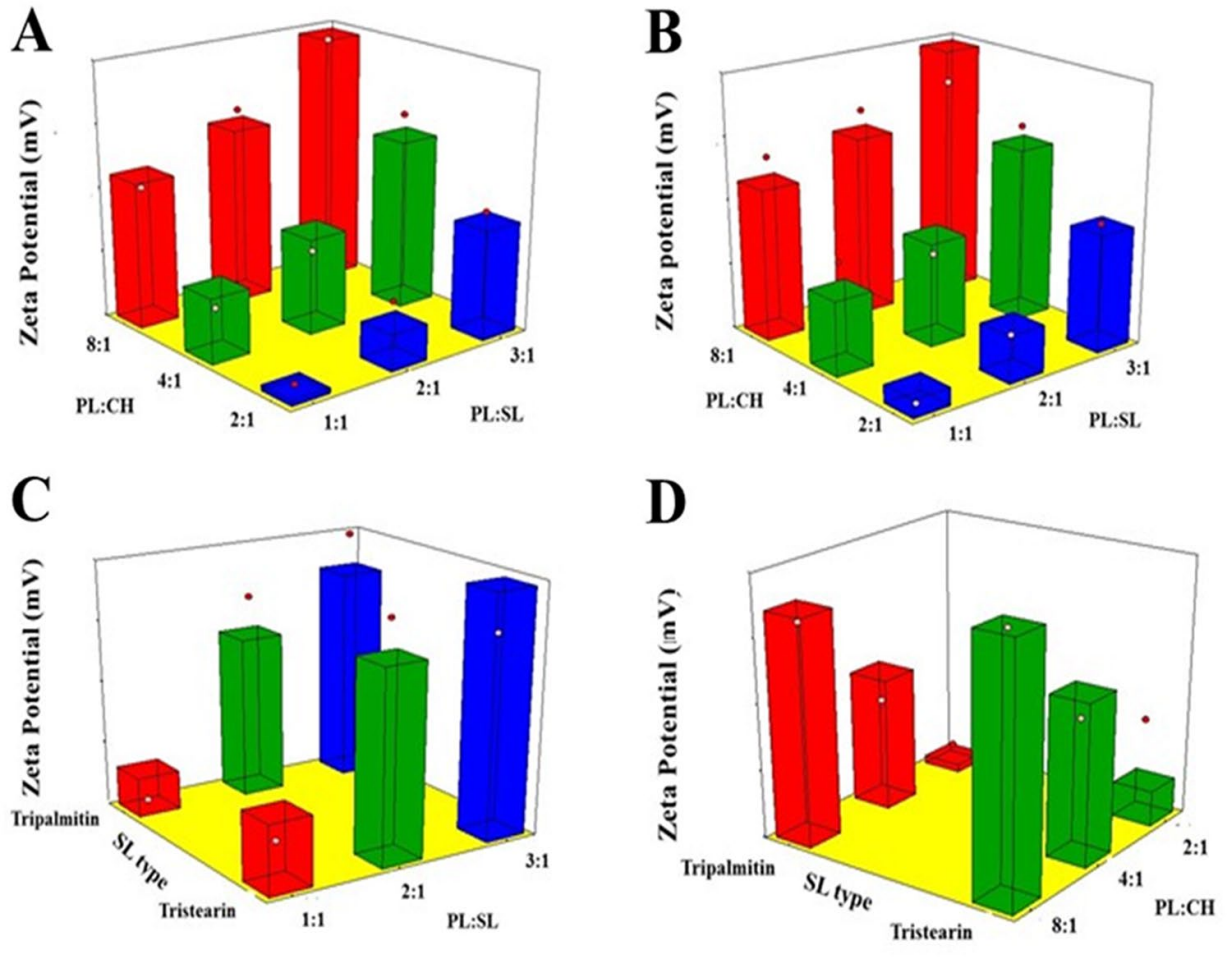
Response 3D surface plot for the influence of PL:SL weight ratio (X_1_), PL:CH molar ratio (X_2_), and SL type (X_3_) on ZP (Y_2_) of VNP emulsomes. (A) SL type (Tripalmitin), (B) SL type (Tristearin), (C) PL:CH (4:1), and (D) PL:SL (2:1).

Figure 2 reports the impact of the investigated variables on VNP emulsomal formulations as 3D-surface plots.

The increase of the PL:CH molar ratio induced a significant increase in the negativity of the emulsomal surface as evidenced by the higher ZP absolute values. This could be related to the increase of the relative amount of the negatively charged PL part of the outer layers (El-Zaafarany et al., 2016; Sudhakar et al., 2016). Tefas et al. described similar higher negative ZP of doxorubicin/curcumin liposomes at higher PL:CH molar ratio (Tefas et al., 2017). The significant reduction of absolute ZP observed at higher CH levels, clearly presented in Figure 2, coincides with the findings reported by Magarkar et al. showing that CH level affects surface charge of lipid membranes in saline solution (reduced sodium ion binding to PL polar head moiety) (Magarkar et al., 2014). Nevertheless, changing SL type did signficantly affect the emulsomal ZP values (*p* = .1410). This result is in agreement with the work carried out by Nayak et al. reporting no marked differences among various SL types in lipid nanoparticulate formulations (Nayak et al., 2010). This observation could be attributed to the existence of the SL in the internal core of vesicles; consequently, they exert no impact on their net surface charge.

### 3.4. Effect on EE% (Y_3_)

**Figure 3.**
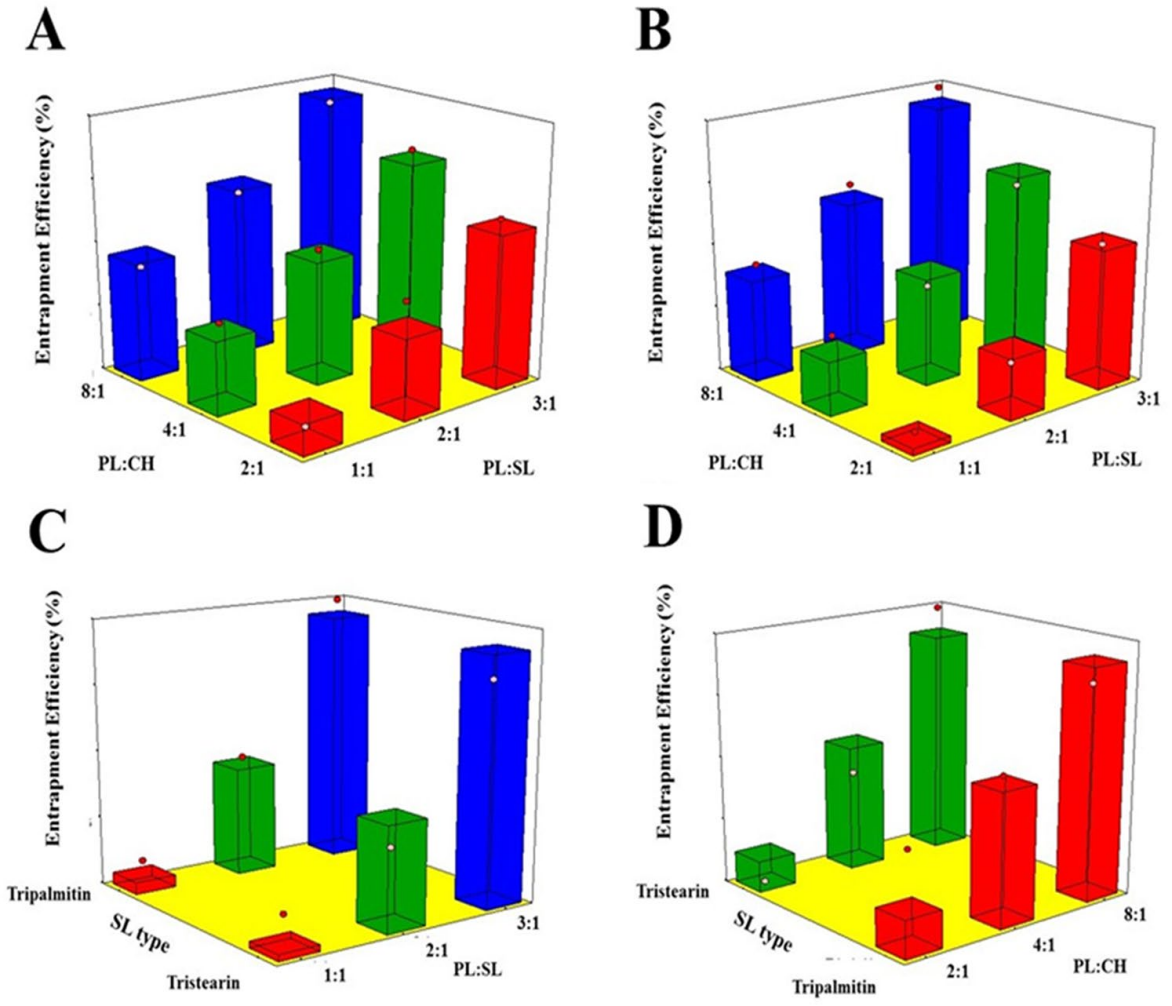
Response 3D surface plot for the influence of PL:SL weight ratio (X_1_), PL:CH molar ratio (X_2_), and SL type (X_3_) on EE% (Y_3_) of VNP emulsomes. (A) SL type (Tripalmitin), (B) SL type (Tristearin), (C) PL:CH (4:1), and (D) PL:SL (2:1).

The entrapment of an active molecule in lipidic vesicular systems could enhance its biological availability as well as provide a controlled release profile (Ong et al., 2016). The proposed formulations showed appreciable drug entrapment exceeding 70%; the average EE% of the proposed formulations are reported in Table 1. As per the ANOVA results, both the linear terms X_1_ and X_2_ corresponding to PL:SL and PL:CH ratio, respectively, exerted a significant influence on the EE% of prepared emulsomes (*p* < .0001). Nevertheless, SL type was not significant (*p* = .694). Figure 3 illustrates the effect of the investigated variables on the EE% of VNP emulsomes in the form of 3D-surface plots.

It was evident that the drug encapsulation increases at higher PL concentrations. Being lipophilic in nature, VNP exhibits more efficient encapsulation in the emulsomal bilayer with increasing PL amount. Thus, increasing PL with respect to SL or CH results in increased PL available to encapsulate the drug within the bilayer (Ahmed et al., 2018). This finding is in line with that of Upadhyay et al. reporting the highest EE at the highest PL:CH ratio of nanoliposomes loaded with an anti-schizophrenic drug (Upadhyay et al., 2017).

Regarding SL type, the absence of differences between the two types may be due to the close similarity between their lipophilicity owing to the close number of carbon atoms in their structure.

### 3.5. Effect on RE_24h_ (Y_4_)

All the formulations exhibited slow drug release during the first 2 h with no observed burst release (data not shown). This finding might be due to the lipophilic nature of the drug that leads to its encapsulation within the lipophilic part of the emulsomes bilayer. Subsequent release of VNP from the proposed formulations was gradual over a period of 24 h, in accordance with previous studies (Tiwari et al., 2019; Aldawsari et al., 2021). RE_24h_ was computed and used for statistical comparison of the prepared formulations (Table 1). Delayed drug release could be deducted by the reduced RE_24h_ values. ANOVA results revealed a significant effect of all the linear terms corresponding to the investigated variables on the RE_24h_. According to the computed *p*-value, the PL:CH exhibited the most significant effect on RE_24h_, followed by SL type, and then PL:SL ratio (*p* = .0001, .0008, and .0018, respectively). Furthermore, the interaction terms X_1_X_3_ and X_2_X_3_, corresponding to the interaction between SL type and either PL:SL ratio or PL:CH, were significant at the 95% level of significance.

**Figure 4.**
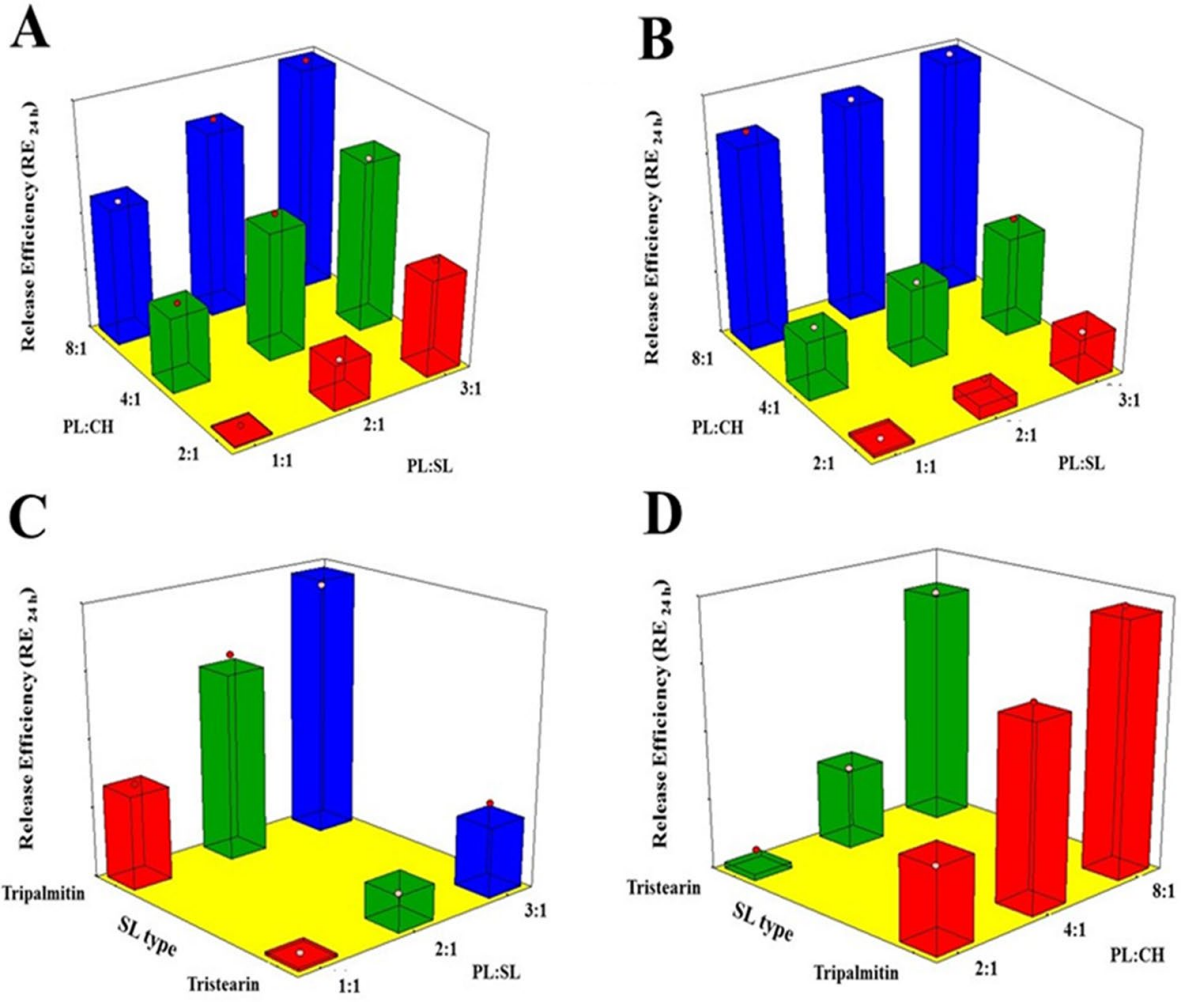
Response 3D surface plot for the influence of PL:SL weight ratio (X_1_), PL:CH molar ratio (X_2_), and SL type (X_3_) on RE_24h_ (Y_4_) of VNP emulsomes. (A) SL type (Tripalmitin), (B) SL type (Tristearin), (C) PL:CH (4:1), and (D) PL:SL (2:1).

Figure 4 reports the effects of the investigated variables on the RE_24h_ of VNP emulsomal formulations as 3D-surface plots.

It was evident that RE_24h_ was positively influenced by the PL:CH ratio. This could be attributed to the enhanced bilayer rigidity with consequent delayed drug release at higher CH proportions. CH has been reported to modify membrane fluidity via the limitation of the hydrocarbon chains mobility; this could in turn lead to reduced bilayer permeability as well as reduced efflux of the entrapped drug leading to slow drug release (Hathout et al., 2007; Sudhakar et al., 2016). Additionally, higher PL content could potentially contribute to increased drug release owing to the bipolar nature of PL that facilitates drug diffusion across lipid bilayer and thus promotes its release (Upadhyay et al., 2017). Furthermore, RE_24h_ was markedly decreased at higher SL amounts. This could depend on the higher entrapment of the lipophilic VNP within the SL core of the emulsomes. Similar drug release pattern was observed for trilaurin-based silybin emulsomes (Zhou & Chen, 2015). With regard to the SL type effect, TS showed better results in slowing down VNP release compared to TP. This could be explained based on the longer chain of TS which requires more time for the drug to diffuse to the release medium, thus resulting in slower release (Sadiq & Rassol, 2014).

### 3.6. Selection of the optimized emulsomal formulation

Selection of the optimized formulation was performed on the basis of the desirability function. The criteria set for the optimization process was minimizing VS and RE_24h_, while maximizing ZP absolute value and EE%. The composition of optimal VNP emulsomes was PL:SL (3:1), PL:CH (2:1), and SL type (TS) (Table 1; Run: **E12**). The combination of the optimized levels is predicted to fulfill the set goals with an overall desirability of 0.718. Thereof, E12, which is prepared at the combination of the optimized levels, was selected for further investigations. The measured responses of the optimized formulation are listed in Table 1.

### 3.7. Surface tailoring of optimal VNP emulsomal formulation

Tailoring of the selected optimal VNP emulsomes surface was performed via either cationization or PEGylation using SA or MPEG-DSPE, respectively, with the aim of enhancing penetration via BBB. The development of cationic emulsomes by using SA allows for electrostatic attraction between the surface positive charge and the negative charges on the BBB resulting in adsorptive-mediated endocytosis and enhanced permeation via BBB (Salem et al., 2015; Vijayakumar et al., 2016). Conversely, the steric stabilization produced by the addition of MPEG-DSPE could result in surpassing opsonization and phagocytosis by the reticuloendothelial system (macrophages), thus, leading to the development of long circulating emulsomes (Nageeb El-Helaly et al., 2017). It is well-known that nanoparticles with a size smaller than 200 nm, representing the estimated limiting size for a nanoparticle to undergo endocytosis through a clathrin-mediated mechanism, are more efficient in crossing the BBB (Betzer et al., 2017); however, the lower ability to cross the BBB of nanoparticles characterized, among all, by a higher size (>200 nm) could be enhanced by surface decoration, allowing to take advantage of transport- and receptor-mediated transcytosis (Lin et al., 2016; Liu et al., 2018), to avoid the endocytotic pathway and deliver the nanoparticle and its cargo directly in the cell cytoplasm (Lindgren et al., 2000), to increase the circulation time, thus leading to an enhancement of the uptake at brain level (Ou et al., 2018).

### 3.8. TEM analysis

**Figure 5.**
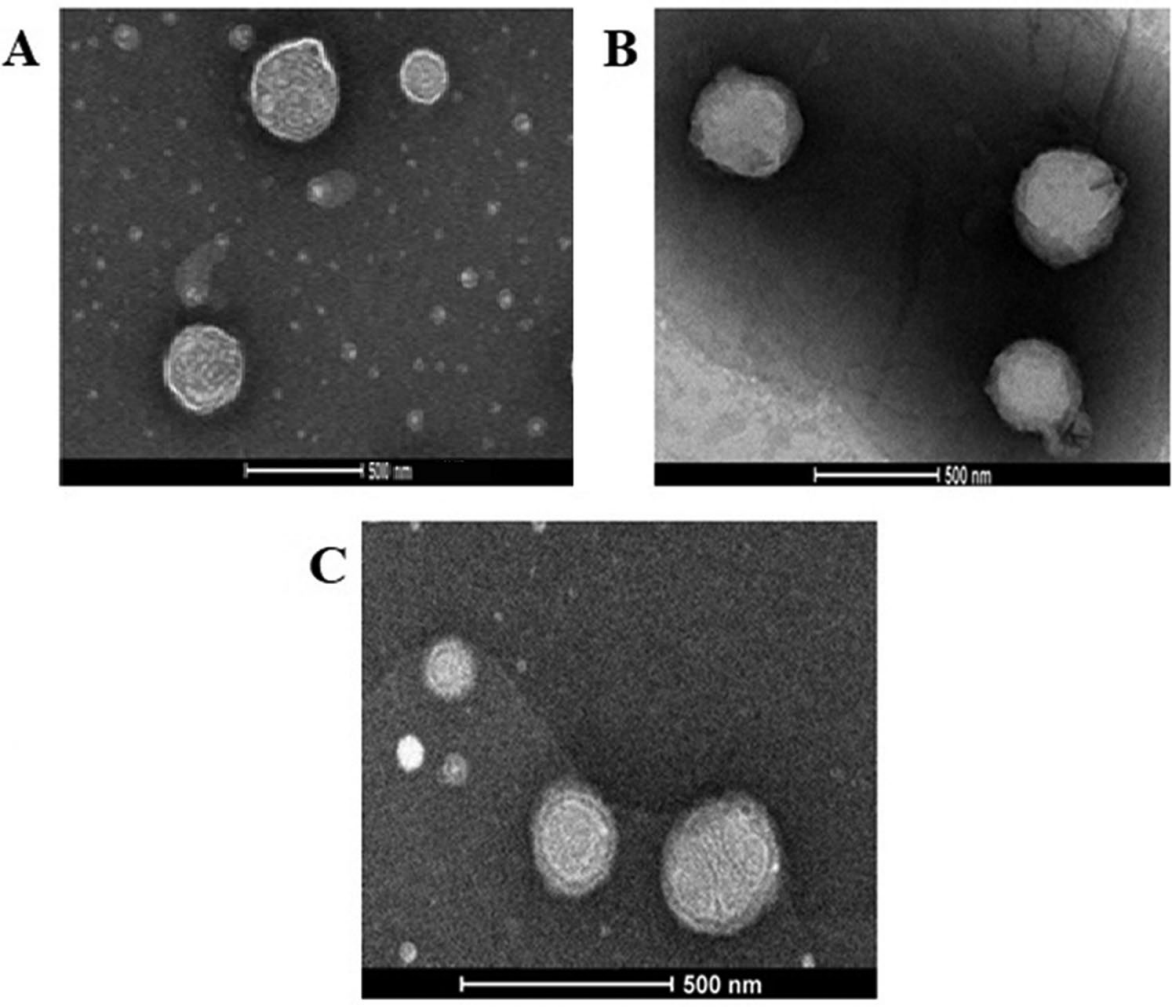
TEM micrographs of (A) optimized VNP emulsomes E12, (B) SA surface-tailored emulsomes E19, and (C) MPEG-DSPE surface-tailored emulsomes E24. Magnification = 22500X.

TEM micrographs showed almost spherical vesicles with uniform size for the optimized VNP emulsomes (E12) and their corresponding surface-tailored formulations. Figure 5(A), depicting E12 formulation, shows PLs as bright ring enclosing the SL core; however, the bright ring was absent in Figure 5(B,C); this difference might be due to the presence of SA or MPEG-DSPE on the vesicular surface (El-Laithy et al., 2011; El-Zaafarany et al., 2016; Mehanna et al., 2017).

The sharp bright contour in Figure 5(A) could therefore be due to the phospholipid bilayer, while the relatively thick ring around the surface of tailored emulsomes could be attributed to the moieties attached to the lipid bilayer to modify the surface characteristics. Additionally, the slight difference between the measured size and that observed with TEM could depend on a possible aggregation process taking place during the processing for imaging.

### 3.9. Characterization of surface-tailored VNP emulsomes

#### 3.9.1. VS assessment

As shown in Table 1, cationic VNP emuslomes were characterized by a larger size compared to the corresponding optimized formulation with no surface tailoring (E12). With regard to increase in size of surface tailored emulsomes using cationic agent, as it has been shown by Carmona-Ribeiro and de Melo Carrasco, cationic agents can contribute to increase the surface charge of nanoemulsions and may also act as preservatives (Carmona-Ribeiro & de Melo Carrasco, 2013). Statistical analysis using one-way ANOVA revealed a marked variation in VS among the optimized emulsomal formulations and their corresponding cationic forms prepared by using different molar concentrations of SA (*p* = .001). Tukey’s HSD test, performed to point out the source of variation, showed a significant variation between the optimized emulsomes (E12) and each of the SA surface-tailored formulations (*p* = .039, .005, and .001 for E19, E20, and E21, respectively). Nevertheless, no significant difference was found when using different concentrations of SA. The marked increase observed in VS of SA surface-tailored emulsomes compared to the corresponding optimized formulation could be attributed to the characteristics of the SA solution. Being viscous in nature, SA solution might influence the emulsomal dispersibility; further and mostly important, it might attract the surface polar heads of the PL bilayer owing to its positive charge. Pushing the surface polar heads of the PLs outwards might result in increased spacing. This observation is supported by the results reported by Narayan et al. (2016) and Mehanna et al. (2017), who found larger SA surface-modified risperidone and ketorolac vesicles compared to the corresponding neutral formulations. On the contrary, PEGylated emulsomes were smaller than the corresponding optimized emulsomes without surface-tailoring, E12, as already reported in Table 1. One-way ANOVA revealed a significant variation among the VS of the optimized emulsomes and the corresponding PEGylated forms (*p* = .002). As per the results of Tukey’s HSD test, the optimized VNP emulsomes differ significantly from the surface-tailored emulsomes prepared at various concentrations of MPEG-DSPE (*p* = .002, .003, and .012, for E22, E23, and E24, respectively); however, the effect of MPEG-DSPE concentration did not significantly influence the size. The marked reduction in the VS upon PEGylation might imply the penetration of the PEGylated polymer into emulsomal bilayer, pressing them together, and thus, enhancing PL packaging within the emulsomes bilayer (Esfahani et al., 2014). Similar size reduction of PEGylated vesicles compared to non-PEGylated was previously reported by Esfahani et al. (2014) and Muppidi et al. (2012).

#### 3.9.2. ZP assessment

The incorporation of surface modifiers is expected to affect the surface charge of the optimized VNP emulsomes. The ZP of the optimized emulsomal formulation shifted from negative to positive charge upon incorporation of SA, as shown in Table 1. One-way ANOVA revealed a significant variation in ZP among the optimized emulsomes (E12) and the corresponding cationic ones (*p* < .001). Tukey’s HSD test showed that E12 differs significantly from the cationic SA surface-tailored emulsomes (*p* < .001 for E19, E20, and E21). However, the difference between different concentrations of SA was not significant. The observed shift in the ZP is attributed to the key role played by the positive charge induction property of SA. Similar positive shift in the ZP upon addition of SA during pramipexole liposomal formulation was reported by Ghule & Bhoyar (2018).

PEGylated VNP emulsomes ZP remains in the negative side, however, the incorporation of MPEG-DSPE led to the reduction of the mean ZP absolute value compared to the optimized non-PEGylated formulation (Table 1). ANOVA revealed a significant difference among the ZP of E12 and the corresponding PEGylated formulation (*p* < .001). Tukey’s HSD test showed that the ZP of each PEGylated emulsomal formulation differs significantly from the corresponding optimized non-PEGylated emulsomes (E12) (*p* = .003, .001, and .001 for E22, E23, and E24, respectively). Additionally, a statistically significant difference (*p* = .023) between E22 and E24, prepared at 0.1 and 0.3 M MPEG-DSPE, respectively, was found. The impact of MPEG-DSPE on ZP could be explained on the basis of the charge-shielding influence of polyethylene glycol moiety (Narayan et al., 2016).

#### 3.9.3. EE% assessment

Although EE% of SA surface-modified emulsomes was greater than that observed for E12, the difference was not statistically significant after ANOVA analysis (*p* = .623). The observed non-significant increase in the EE% might be due to the increased spacing in the PL bilayer, caused by the electrostatic interaction between positively charged SA and negatively charged polar head groups, with consequent increase in the entrapping volume within the lipid bilayer. However, it was observed that the rise in SA concentration above 0.5 M caused decreased drug entrapment. This could be attributed to the increased repulsion between PL layers at higher SA concentrations, resulting in distortion of emulsomes structure and formation of unstable vesicles. These findings are in agreement with previous research studies reporting similar effects for both SA addition and SA variation concentration on EE vesicular systems (Vyas et al., 2010; Mehanna et al., 2017).

Regarding MPEG-DSPE surface-tailored emulsomes, ANOVA revealed a significant difference in the EE% among the optimized emulsomes, E12, and the surface modified ones (*p* = .004). Tukey’s HSD test revealed that E12 differs significantly from each of the surface-modified emulsomes prepared at 0.2 and 0.3 M of the PEGylated polymer (*p* = .039 and .005 for E23 and E24, respectively). Furthermore, a statistically significant difference was found between E22, prepared at 0.1 M, and E24, prepared at 0.3 M (*p* = .019). The increased drug entrapment of MPEG-DSPE surface-tailored emulsomes in comparison to the non-PEGylated optimized formulation might be due to the existence of PEG moieties on the emulsomal external surface that leads to vesicular stabilization and reduced VNP leakage. Additionally, incorporation of the PEGylated polymer could result in higher lipid amount available for better entrapment of the lipophilic drug. This result is in line with those reported by Sufhakar et al. that observed the enhanced entrapment of ritonavir within PEGylated liposomes (Sudhakar et al., 2016).

#### 3.9.4. In vitro drug release

**Figure 6.**
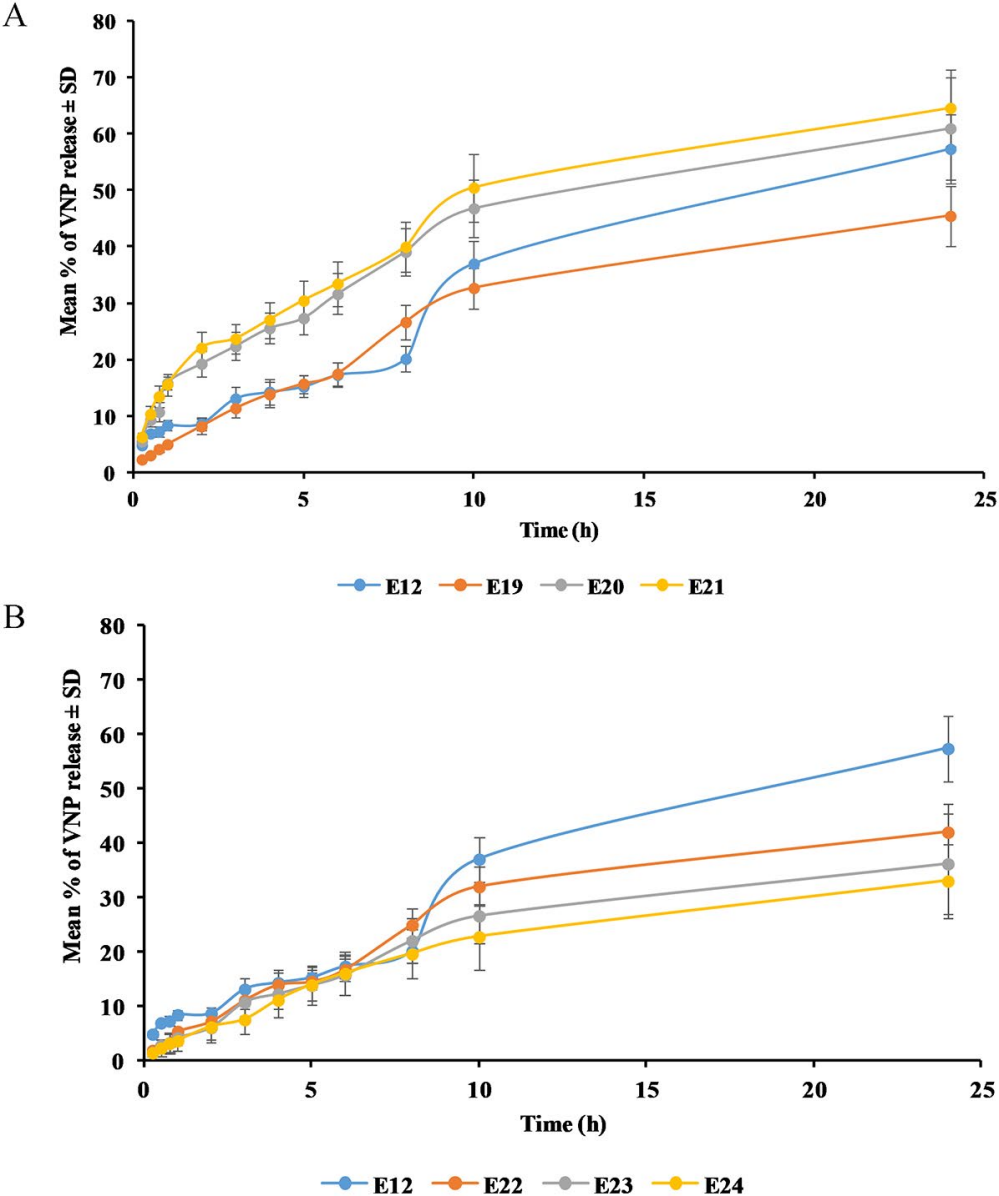
Release profile of (A) SA and (B) MPEG-DSPE surface-tailored emulsomes in PBS (pH 6.8) at 35 ± 0.5 °C compared to the optimized emulsomes (E12). Results are presented as mean (*n* = 3) ± *SD.*

Regarding SA surface-tailored emulsomes, it was observed that the addition of SA up to 0.25 M caused the delay of VNP release. However, increasing molar concentration of SA above 0.25 M led to faster drug release (Figure 6(A)).

One-way ANOVA revealed a significant difference in the RE_24h_ among the optimized formulation, E12, and the cationic emulsomes (*p* = .035). With the aim to point out the difference source Tukey’s HSD test was performed revealing that E12 does not differ significantly from any of its corresponding cationic emulsomes. Nevertheless, a significant difference was observed between E19, prepared at 0.25 M, and E21, prepared at 0.75 M (*p* = .032). The observed drug release delay upon addition of 0.25 M of SA could be explained based on the electrostatic attraction between the anionic drug and the cationic emulsomes. This interaction could result in confinement of the drug within the emulsomes with consequent reduced release. However, the observed faster release at higher SA concentrations could be explained on the basis of the previously described emulsomal structure disruption and reduced drug entrapment. Delayed release from positively charged vesicles compared to the neutral and anionic forms has been previously reported in different studies (Narayan et al., 2016; Upadhyay et al., 2017; Aldawsari et al., 2021).

Compared to the optimizied formulation E12, MPEG-DSPE surface-tailored emulsomes showed lower RE_24h_ (Figure 6(B)). One-way ANOVA revealed a significant difference among E12 and the corresponding PEGylated formulations (*p* = .001). Tukey’s HSD test showed that RE_24h_ of each of PEGylated emulsome formulations differs significantly from that of the optimized formulation (*p* = .007, .003, and .001 for E22, E23, and E24, respectively). Conversely, no significant effect was revealed for the concentration of MPEG-DSPE. The reported delay of VNP release upon addition of MPEG-DSPE might be due to the increased total amount of lipids available, thus, enhancing the encapsulation as well as the confinement of the lipophilic VNP within the formulation, also controlling its release. Similar pattern for drugs’ release from PEGylated vesicular systems have been described by Sudhakar et al. (2016) and Tsermentseli et al. (2018).

The cationic and PEGylated VNP emulsomes (E19 and E24, respectively) characterized by highest EE% and ZP magnitude, and lowest VS and RE_24h_ were selected for further *in vivo* assessment.

### 3.10. Pharmacokinetic assessment of VNP emulsomes

**Figure 7.**
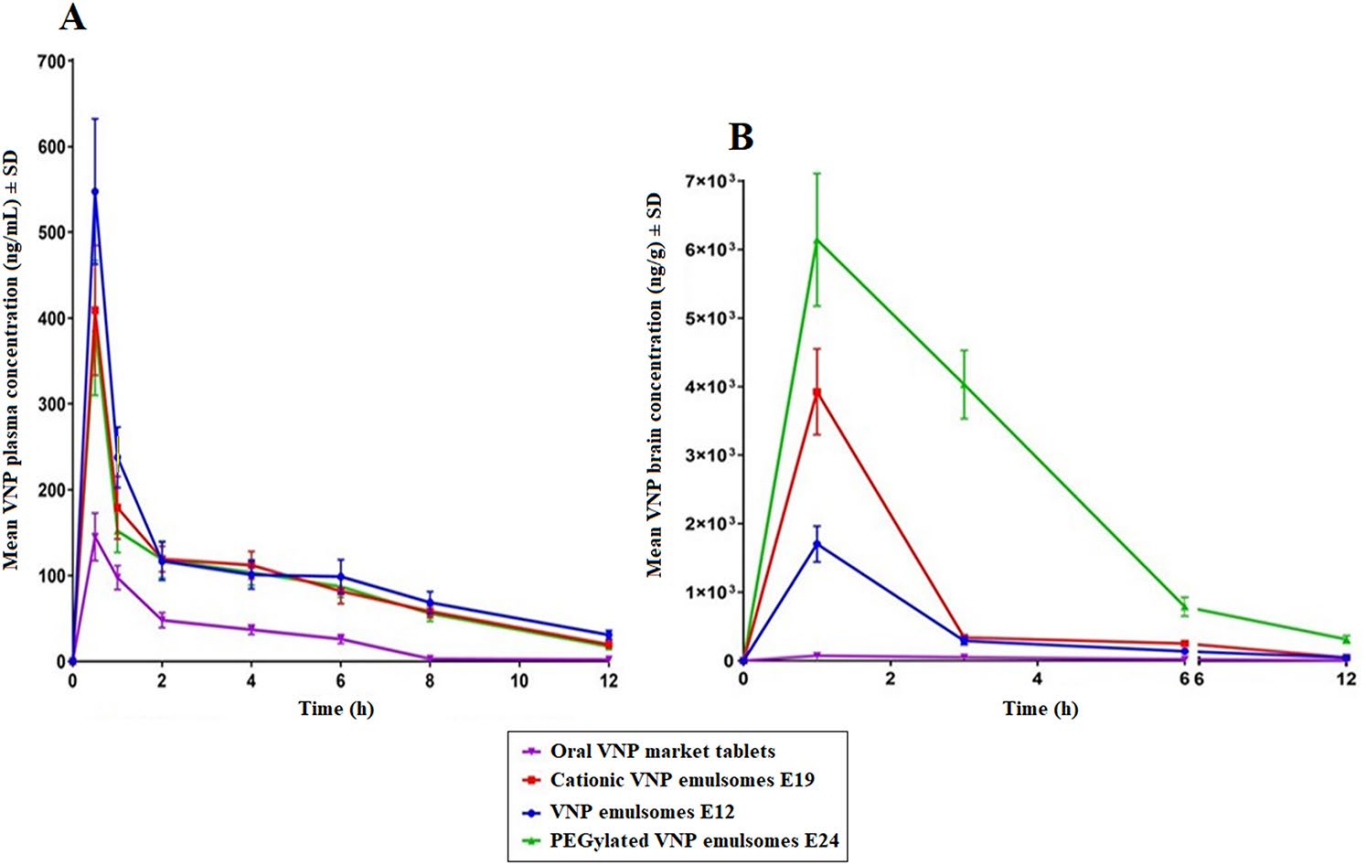
Mean VNP concentration in (A) plasma and (B) brain following the intranasal administration of VNP optimized emulsomes (E12), SA surface-tailored emulsomes (E19) and MPEG-DSPE surface-tailored emulsomes (E24) compared to the orally administered market tablets in rats. Results are presented as mean (*n* = 6) ± *SD.*

Intranasal instillation was performed as a single shot of 0.375 mL in each nostril using polyethylene tubes. Mean VNP concentrations in plasma (Figure 7(A)) and brain (Figure 7(B)) following the intranasal administration of E12, E19, and E24 are shown in **Figure 7**.

The determined pharmacokinetic parameters, namely maximum plasma concentration (*C*_max_) and the time needed to reach it (*T*_max_), in addition to the computed area under the plasma-concentration time curve (AUC_0-12_ and AUC_0-∞_) are reported in Table 3.

**Table 3.** Measured plasma and brain pharmacokinetic parameters of VNP following the intranasal administration of E12, E19, and E24 in comparison with the orally administered market tablets.

	Plasma				Brain			
	*T*_max_ (h)	*C*_max_ (ng/mL)	AUC_(0-12)_ (ng/mL/h)	AUC_(0-∞)_ (ng/mL/h)	*T*_max_ (h)	*C*_max_ (ng/g)	AUC_(0-12)_ (ng/mL/h)	AUC_(0-∞)_ (ng/mL/h)
**E12**	0.50	547.32 ± 84.53	1293.11 ± 80.43	1492.51 ± 89.60	1.00	1721.41 ± 242.72	4041.70 ± 578.24	4260.97 ± 621.69
**E19**	0.50	409.05 ± 75.65	1118.87 ± 75.28	1225.85 ± 91.00	1.00	3924.67 ± 627.65	8003.63 ± 1204.84	8195.01 ± 1215.45
**E24**	0.50	388.46 ± 78.37	1069.27 ± 124.12	1164.86 ± 123.64	1.00	6144.50 ± 968.86	23751.48 ± 1445.82	24914.79 ± 1523.30
**Oral VNP**	1.00	145.03 ± 27.79	355.91 ± 25.19	361.17 ± 24.63	1.00	73.24 ± 7.79	333.69 ± 31.89	342.07 ± 34.48

Abbreviations: VNP: vinpocetine; *C*_max_: maximum plasma concentration; *T*_max_: time needed to reach the maximum plasma concentration; AUC_0-12_: area under the plasma concentration time curve from 0 to 12 h; AUC_0-∞_: area under the plasma concentration time curve from 0 to ∞. Results are presented as mean ± *SD* (*n* = 6) for *C*_max_, AUC_0-12_, and AUC_0-∞_ and median (*n* = 6) for *T*_max_.

#### 3.10.1. Plasma pharmacokinetics

It is clear that higher VNP plasma levels were achieved upon intranasal administration of the emulsomal formulations compared to oral administration of the market product (higher *C*_max_ and AUC) (Figure 7(A) and Table 3). ANOVA showed that the difference between *C*_max_, AUC_0-12_, and AUC_0-∞_ was significant among the tested formulations (*p* < .0001). Tukey’s HSD test revealed that the aforementioned parameters were significantly higher for intranasal emulsomes in comparison to the oral market product (*p* < .0001). This could be attributed to the bypass of first pass metabolism (hepatic) as a consequence of the intranasal administration. However, both SA and MPEG-DSPE surface-tailored emulsomes showed significantly lower *C*_max_, AUC_0-12_, and AUC_0-∞_ compared to the optimized surface nontailored emulsomes at 95% level of significance, suggesting the possible ability of the surface modification to boost drug availability at brain rather than plasma level. Regarding *T*_max_, all the emulsomal formulations reached the highest plasma concentration after 0.5 h compared to 1 h needed for the market product. However, no statistical difference was found among *T*_max_ of the formulations as per Kruskal–Wallis test results (*p* = .467). The lower *T*_max_ of the emulsomes could be attributed to the rich vasculature of the nasal cavity, that allows for faster absorption compared to the oral route (Abdel-Mottaleb & Lamprecht, 2011; Khan et al., 2017).

#### 3.10.2. Brain pharmacokinetics

Figure 7(B**)** shows higher *C*_max_, AUC_0-12_, and AUC_0-∞_ for the intranasal emulsomal formulations compared to the market product. All formulations reached the maximum brain concentration 1 h after administration. As per ANOVA results, a statistical significant difference in the *C*_max_, AUC_0-12_, and AUC_0-∞_ among the tested formulations was observed (*p* < .0001). Tukey’s HSD test revealed that the aforementioned parameters were significantly higher following intranasal administration of emulsomes compared with oral administration of the market product, (*p* ≤ .05). Additionally, the multiple comparison revealed significantly higher parameters for each of the surface-tailored emulsomal formulations compared to the corresponding surface non-tailored emulsomes (*p* < .001).

The higher brain levels of VNP achieved by the nasal emulsomes could be attributed to the uptake of VNP emulsomes via either the systemic or olfactory pathways. Unlike the oral route, the nasal one could achieve direct absorption of VNP into the systemic circulation bypassing the pre-systemic metabolism, thus more drug is available to reach the brain. Additionally, the olfactory pathway allows for drug uptake directly from the nasal cavity into cerebrospinal fluid and brain tissues (Ahirrao & Shrotriya, 2017; Liu et al., 2018). Another factor that should be considered is the high lipophilicity of the lipid-rich emuslomes that may boost the systemic absorption of VNP as well as enhance its uptake via BBB (Arumugam et al., 2008; Al Asmari et al., 2016; Liu & Ho, 2017). The above findings are in agreement with several research studies reporting the improvement in the availability of drugs at brain level following the intranasal administration compared to the oral route (Al Asmari et al., 2016; Ahirrao & Shrotriya, 2017; Liu et al., 2018).

The higher VNP uptake into the brain coming from the administration of SA surface-tailored emulsomes could be the consequence of the electrostatic interaction between cationic emulsomes and negative charges on the BBB, allowing for higher penetration into the brain (Ghule & Bhoyar, 2018). Conversely, the higher brain levels achieved by the MPEG-DSPE surface-tailored emulsomes could be ascribed to the high binding affinity of PEG moieties to the carrier-meditated glucose transporter, GLUT1; glucose transporters are highly expressed in the brain capillary endothelial cells and have the responsibility of glucose transportation into or out of the brain to maintain the cerebral function. Accordingly, binding of PEG to GLUT facilitates delivery to the brain (Xie et al., 2012; Caruso et al., 2019).

The *in vivo* study confirmed the ability of the surface-tailored emulsomes to achieve improved VNP brain delivery compared to either oral market product or corresponding non-tailored formulations. PEG is able to target the brain endothelial cell receptors via induction of receptor-mediated transcytosis (Qiao et al., 2012; Pulgar, 2018), while SA targets the brain via the interaction with the negative charges existing on the BBB (Bors & Erdő, 2019; Satapathy et al., 2021), resulting in adsorptive-mediated endocytosis.

## 4. Conclusions

The present work demonstrated the successfulness of the proposed intranasal administration of VNP emulsomes in enhancing both plasma and brain levels of VNP. Full factorial design was successfully applied for the optimization of VNP emulsomes. The selected formulation, indicated as E12, exhibited minimized VS, maximized absolute ZP, and drug entrapment as well as controlled drug release. Surface tailoring of the optimized emulsomal formulation was successfully achieved via either cationization or PEGylation. All the considered new formulations demonstrated an enhanced ability, in terms of VNP plasma and brain concentrations, compared to the market option. In conclusion, surface-tailored intranasal emulsomes could represent a promising platform to enhance drugs delivery to the brain.

## Patent

The present work is protected under the Patent No.:US11058637B1, United States Patent and Trademark Office (USPTO).
